# Estimating the Unreported Number of Novel Coronavirus (2019-nCoV) Cases in China in the First Half of January 2020: A Data-Driven Modelling Analysis of the Early Outbreak

**DOI:** 10.3390/jcm9020388

**Published:** 2020-02-01

**Authors:** Shi Zhao, Salihu S. Musa, Qianying Lin, Jinjun Ran, Guangpu Yang, Weiming Wang, Yijun Lou, Lin Yang, Daozhou Gao, Daihai He, Maggie H. Wang

**Affiliations:** 1 JC School of Public Health and Primary Care, Chinese University of Hong Kong, Hong Kong 999077, China; maggiew@cuhk.edu.hk; 2 Shenzhen Research Institute of Chinese University of Hong Kong, Shenzhen 518060, China; 3 Department of Applied Mathematics, Hong Kong Polytechnic University, Hong Kong 999077, China; salihu-sabiu.musa@connect.polyu.hk (S.S.M.); yijun.lou@polyu.edu.hk (Y.L.); 4 Michigan Institute for Data Science, University of Michigan, Ann Arbor, Michigan, MI 48104, USA; qianying@umich.edu; 5 School of Public Health, Li Ka Shing Faculty of Medicine, University of Hong Kong, Hong Kong 999077, China; jimran@connect.hku.hk; 6 Department of Orthopaedics and Traumatology, Chinese University of Hong Kong, Hong Kong 999077, China; kennethgpy@link.cuhk.edu.hk; 7 SH Ho Scoliosis Research Lab, Joint Scoliosis Research Center of Chinese University of Hong Kong and Nanjing University, Hong Kong 999077, China; 8 School of Mathematics and Statistics, Huaiyin Normal University, Huaian 223300, China; 9 School of Nursing, Hong Kong Polytechnic University, Hong Kong 999077, China; l.yang@polyu.edu.hk; 10 Department of Mathematics, Shanghai Normal University, Shanghai 200234, China; dzgao@shnu.edu.cn

**Keywords:** novel coronavirus, outbreak, modelling, underreporting, reproduction number, China

## Abstract

Background: In December 2019, an outbreak of respiratory illness caused by a novel coronavirus (2019-nCoV) emerged in Wuhan, China and has swiftly spread to other parts of China and a number of foreign countries. The 2019-nCoV cases might have been under-reported roughly from 1 to 15 January 2020, and thus we estimated the number of unreported cases and the basic reproduction number, *R*_0_, of 2019-nCoV. Methods: We modelled the epidemic curve of 2019-nCoV cases, in mainland China from 1 December 2019 to 24 January 2020 through the exponential growth. The number of unreported cases was determined by the maximum likelihood estimation. We used the serial intervals (SI) of infection caused by two other well-known coronaviruses (CoV), Severe Acute Respiratory Syndrome (SARS) and Middle East Respiratory Syndrome (MERS) CoVs, as approximations of the unknown SI for 2019-nCoV to estimate *R*_0_. Results: We confirmed that the initial growth phase followed an exponential growth pattern. The under-reporting was likely to have resulted in 469 (95% CI: 403–540) unreported cases from 1 to 15 January 2020. The reporting rate after 17 January 2020 was likely to have increased 21-fold (95% CI: 18–25) in comparison to the situation from 1 to 17 January 2020 on average. We estimated the *R*_0_ of 2019-nCoV at 2.56 (95% CI: 2.49–2.63). Conclusion: The under-reporting was likely to have occurred during the first half of January 2020 and should be considered in future investigation.

## 1. Introduction

A novel coronavirus (2019-nCoV) infected pneumonia infection, which is deadly [1], was first identified in Wuhan, China in December 2019 [2]. The virus causes a range of symptoms including fever, cough, and shortness of breath [3]. The cumulative number of reported cases slowly increased to cumulative 41 cases by 1 January 2020, and rapidly increased after 16 January 2020. As of 26 January 2020, the still ongoing outbreak had resulted in 2066 (618 of them are in Wuhan) confirmed cases and 56 (45 of them were in Wuhan) deaths in mainland China [4], and sporadic cases exported from Wuhan were reported in Thailand, Japan, Republic of Korea, Hong Kong, Taiwan, Australia, and the United States, please see the World Health Organization (WHO) news release via https://www.who.int/csr/don/en/ from 14 to 21 January 2020. Using the number of cases exported from Wuhan to other countries, a research group at Imperial College London estimated that there had been 4000 (95%CI: 1000–9700) cases in Wuhan with symptoms onset by 18 January 2020, and the basic reproduction number (*R*_0_) was estimated at 2.6 (95%CI: 1.5–3.5) [5]. Leung et al. drew a similar conclusion and estimated the number of cases exported from Wuhan to other major cities in China [6], and the potentials of travel related risks of disease spreading was also indicated by [7].

## 2. Objectives and Methods

Due to an unknown reason, the cumulative number of cases remained at 41 from 1 to 15 January 2020 according to the official report, i.e., no new case was reported during these 15 days, which appears inconsistent with the following rapid growth of the epidemic curve since 16 January 2020. We suspect that the 2019-nCoV cases were under-reported roughly from 1 to 15 January 2020. In this study, we estimated the number of unreported cases and the basic reproduction number, *R*_0_, of 2019-nCoV in Wuhan from 1 to 15 January 2020 based on the limited data in the early outbreak. 

The time series data of 2019-nCoV cases in mainland China were initially released by the Wuhan Municipal Health Commission from 10 to 20 January 2020 [8], and later by the National Health Commission of China after 21 January 2020 [9]. The case time series data in December 2019 were obtained from a published study [3]. All cases were laboratory confirmed following the case definition by the national health commission of China [10]. We chose the data up to 24 January 2020 instead of to the present study completion date. Given the lag between timings of case confirmation and news release of new cases, the data of the most recent few days were most likely to be tentative, and thus they were excluded from the analysis to be consistent.

We suspected that there was a number of cases, denoted by *ξ*, under-reported from 1 to 15 January 2020. The cumulative total number of cases, denoted by *C_i_*, of the *i*-th day since 1 December 2019 is the summation of the cumulative reported, *c_i_*, and cumulative unreported cases, *Ξ_i_*. We have *C_i_* = *c_i_* + *Ξ_i_*, where *c_i_* is observed from the data, and *Ξ_i_* is 0 for *i* before 1 January and *ξ* for *i* after 15 January 2020. Following previous studies [11,12], we modelled the epidemic curve, i.e., the *C_i_* series, as an exponential growing Poisson process. Since the data from 1 to 15 January 2020 appeared constant due to unclear reason(s), we removed these data from the fitting of exponential growth. The *ξ* and the intrinsic growth rate (*γ*) of the exponential growth were to be estimated based on the log-likelihood, denoted by *ℓ*, from the Poisson priors. The 95% confidence interval (95% CI) of *ξ* was estimated by the profile likelihood estimation framework with cutoff threshold determined by a Chi-square quantile [13], *χ*^2^_pr__=__0.95, df__=__1_. With *γ* estimated, the basic reproduction number could be obtained by *R*_0_ = 1/*M*(−*γ*) with 100% susceptibility for 2019-nCoV presumed at this early stage. Here, the function *M*(∙) was the Laplace transform, i.e., the moment generating function, of the probability distribution for the serial interval (SI) of the disease [11,14], denoted by *h*(*k*) and *k* is the mean SI. Since the transmission chain of 2019-nCoV remained unclear, we adopted the SI information from Severe Acute Respiratory Syndrome (SARS) and Middle East Respiratory Syndrome (MERS), which share the similar pathogen as 2019-nCoV [15,16,17]. We modelled *h*(*k*) as Gamma distributions with mean of 8.0 days and standard deviation (SD) of 3.6 days by averaging the SI mean and SD of SARS, mean of 7.6 days and SD of 3.4 days [18], and MERS, mean of 8.4 days and SD of 3.8 days [19]. 

We were also interested in inferring the patterns of the daily number of cases, denoted by *ε_i_* for the *i*-th day, and thus it is obviously that *C_i_* = *C_i_*_−1_ + *ε_i_*. A simulation framework was developed for the iterative Poisson process such that **E**[*ε_i_*] = *C_i_*_−1_ × [exp(*γ*) − 1], where function **E**[∙] denoted the expectation. The simulation was implemented starting from 1 January 2020 with a cumulative number of cases seed of 40, the same as reported on 31 December 2019. We conducted 1000 samples and calculated the median and 95% CI. 

## 3. Results and Discussion

**Figure 1:**
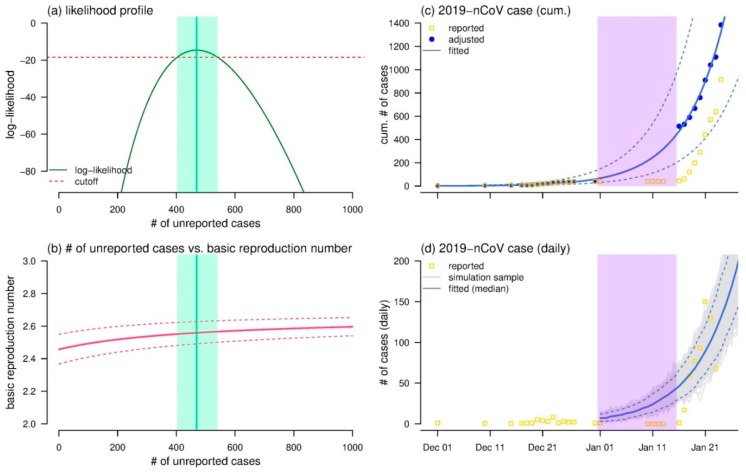
The estimates of the unreported cases between 1 and 15 January 2020, the basic reproduction number (*R*_0_), and fitting results of the number of 2019-nCoV cases time series. Panel (**a**) shows the likelihood profile (*ℓ*, dark green curve) of the estimated number of unreported cases (*ξ*), and the cutoff threshold (horizontal red dashed line) for the 95% CI. The relationship between the number of unreported cases (*ξ*) and *R*_0_, where the bold curve is the mean estimation, and the dashed curves are the 95% CI of estimated *R*_0_. In panels (**a**,**b**), the green shading area represents the 95% CI (on the horizontal axis), and the vertical green line represents the maximum likelihood estimate (MLE) of the number of unreported cases. With the MLE of *R*_0_ at 2.56, panels (**c**,**d**) show the exponential growth fitting results of the cumulative number of cases (*C_i_*) and the daily number of cases (*ε_i_*) respectively. In panels (**c**,**d**), the gold squares are the reported cases, the blue bold curve represents the median of the fitting results, the dashed blue curves are the 95% CI of the fitting results, and the purple shading area represents the time window from 1 to 15 January 2020. In panel (**c**), the blue dots are the cumulative total, i.e., reported and unreported, number of cases. In panel (**d**), the grey curves are the 1000 simulation samples.

The number of 2019-nCoV unreported cases was estimated at 469 (95% CI: 403–540), see Figure 1a, which was significantly larger than 0. This finding implied the occurrence of under-reporting between 1 and 15 January 2020. After accounting for the effect of under-reporting, the *R*_0_ was estimated at 2.56 (95% CI: 2.49–2.63), see Figure 1b, which is consistent with many existing online preprints with range from 2 to 4 [5,20,21,22]. With the *R*_0_ of 2.56 and *ξ* of 469, the exponential growing framework fitted the cumulative total number of cases (*C_i_*) remarkably well, see Figure 1c, referring to McFadden’s pseudo-*R*-squared of 0.99.

Our estimation of *R*_0_ rely on the SI of 2019-nCoV, which remains unknown as of 26 January 2020. In this work, we employed the SIs of SARS and MERS as approximations to that of 2019-nCoV. The determination of SI requires the knowledge of the chain of disease transmission that needs a sufficient number of patient samples and periods of time for follow-up [23], and thus this is unlikely to be achieved shortly. However, using SIs of SARS and MERS as approximation could provide an insight into the transmission potential of 2019-nCoV at the early outbreak. We note that slightly varying the mean and SD of SI would not affect our main conclusions. The *R*_0_ of 2019-nCoV was estimated at 2.56 (95% CI: 2.49–2.63), and it is generally in line with those of SARS, i.e., 2–5 [19,24,25], and MERS, i.e., 2.7–3.9 [26]. 

For the simulated daily number of cases (*ε_i_*), see Figure 1d, we found that *ε_i_* matched the observed daily number after 17 January 2020, but was significantly larger than the observations from 1 to 17 January 2020. This finding implied that under-reporting was likely to have occurred in the first half of January 2020. We estimated that the reporting rate after 17 January 2020 increased 21-fold (95% CI: 18–25) compared to the situation from 1 to 17 January 2020 on average. One of the possible reasons was that the official diagnostic protocol was released by WHO on 17 January 2020 [27], and the diagnosis and reporting efforts of 2019-nCoV infections probably increased. Thereafter, the daily number of newly reported cases started increasing rapidly after 17 January 2020, see Figure 1d. We conducted additional sensitivity analysis by varying the starting date of the under-reporting time window, e.g., 1 January 2020 in the main results, from 2 December 2019 to 3 January 2020, and we report our estimates largely hold. The exact value of the reporting rate was difficult to determine due to lack of serological surveillance data. The reporting rate can be determined if serological surveillance data are available for a population; we would know who was infected (seropositive) and who was not (seronegative), with high confidence. The reporting rate is the ratio of reported cases over the number of seropositive individuals. It was statistically evident that increasing in reporting was likely, and thus it should be considered in the future investigation of this outbreak.

Previous preprint suggested cumulative cases of 1723 (95% CI: 427–4471) as of 12 January 2020, and 4000 (95% CI: 1000–9700) as of 18 January 2020 based on the aggregated international export cases [5]. Our analysis yielded cumulative cases of 280 (95% CI: 128–613) as of 12 January 2020, and 609 (95% CI: 278–1333) as of 18 January 2020 based on the exponential growing mechanistic in the early outbreak. Although our estimate case number appeared to have a lower mean than those estimated by Imai et al. [5], they are not statistically different. This study applied a different screening effort to detect the 2019-nCoV cases from that in Imai et al. [5]. Imai et al. assumed the average screening effort at overseas airports that covered travelers arriving from Wuhan. Whereas we assumed a constant screening effort applied in Wuhan at the same point of time, and then a number of cases (i.e., *ξ*) should have been reported yet failed to be reported in the first half of January 2020 due to all sorts of reasons. It is not surprising that different assumptions yielded different results, and this difference in screening effort also partly explained why the detected cases out of China mainly presented mild symptoms. Thus, it was reasonable that our estimates appeared lower than those estimated by Imai et al. [5]. It must be emphasized that such a gap in the knowledge would be resolved by serological survey study (for a large population to approximate the actual positive rate) or an explicit estimation of the actual reporting rate. 

## 4. Conclusions

Under-reporting was likely to have occurred and resulted in 469 (95% CI: 403–540) unreported cases from 1 to 15 January 2020. The reporting rate after 17 January 2020 was likely to have increased 21-fold (95% CI: 18–25) compared with the situation from 1 to 17 January 2020 on average, and it should be considered in future investigation. We estimated the *R*_0_ at 2019-nCoV to be 2.56 (95% CI: 2.49–2.63).
